# Multimodal Communication in Aphasia: Perception and Production of Co-speech Gestures During Face-to-Face Conversation

**DOI:** 10.3389/fnhum.2018.00200

**Published:** 2018-06-14

**Authors:** Basil C. Preisig, Noëmi Eggenberger, Dario Cazzoli, Thomas Nyffeler, Klemens Gutbrod, Jean-Marie Annoni, Jurka R. Meichtry, Tobias Nef, René M. Müri

**Affiliations:** ^1^Perception and Eye Movement Laboratory, Department of Neurology and Clinical Research, University of Bern Inselspital, Bern, Switzerland; ^2^Donders Centre for Cognitive Neuroimaging, Donders Institute for Brain, Cognition and Behaviour, Radboud University, Nijmegen, Netherlands; ^3^Gerontechnology and Rehabilitation Group, ARTORG Center for Biomedical Engineering Research, University of Bern, Bern, Switzerland; ^4^Center of Neurology and Neurorehabilitation, Luzerner Kantonsspital, Luzern, Switzerland; ^5^University Neurorehabilitation Clinics, Department of Neurology, University of Bern Inselspital, Bern, Switzerland; ^6^Neurology Unit, Laboratory for Cognitive and Neurological Sciences, Department of Medicine, Faculty of Science, University of Fribourg, Fribourg, Switzerland

**Keywords:** aphasia, gesture, eye-tracking, gaze, conversation, lesion mapping

## Abstract

The role of nonverbal communication in patients with post-stroke language impairment (aphasia) is not yet fully understood. This study investigated how aphasic patients perceive and produce co-speech gestures during face-to-face interaction, and whether distinct brain lesions would predict the frequency of spontaneous co-speech gesturing. For this purpose, we recorded samples of conversations in patients with aphasia and healthy participants. Gesture perception was assessed by means of a head-mounted eye-tracking system, and the produced co-speech gestures were coded according to a linguistic classification system. The main results are that meaning-laden gestures (e.g., iconic gestures representing object shapes) are more likely to attract visual attention than meaningless hand movements, and that patients with aphasia are more likely to fixate co-speech gestures overall than healthy participants. This implies that patients with aphasia may benefit from the multimodal information provided by co-speech gestures. On the level of co-speech gesture production, we found that patients with damage to the anterior part of the arcuate fasciculus showed a higher frequency of meaning-laden gestures. This area lies in close vicinity to the premotor cortex and is considered to be important for speech production. This may suggest that the use of meaning-laden gestures depends on the integrity of patients’ speech production abilities.

## Introduction

Co-speech gestures are omnipresent during face-to-face interaction. At the same time, co-speech gestures also occur when the interaction partner is not visually present, e.g., when people are talking on the phone. This implies that co-speech gestures do not only convey communicative meaning (McNeill, 1992), but also support speech production by facilitating lexical retrieval (Rauscher et al., 1996; Krauss and Hadar, 1999). Historically, a long-standing debate concerns the question whether speech and gestures are based on a unitary communication system (McNeill, 1992; Kendon, 2004), or on separate—though tightly interacting—communication systems (Levelt et al., 1985; Hadar et al., 1998b). More recently it has been discussed how tightly speech and gesture are integrated (Kita et al., 2017). According to the Sketch Model (De Ruiter, 2000), which has been built upon Levelt’s model of speech production (Levelt, 1989), speech and gesture originate from a shared communicative intention, but are produced via separate channels.

Aphasia is an acquired language disorder that results from a brain lesion to the language-dominant hemisphere (Damasio, 1992; Dronkers et al., 2004; Butler et al., 2014). The disorder generally affects different modalities, i.e., speaking, understanding, reading and writing. Nonverbal communication, such as co-speech gestures, may help patients to express themselves more intelligibly. Moreover, the investigation of gesture processing in aphasia allows to gain new insights into the neurocognitive underpinnings of speech and gesture processing. Previous research in this field mainly focused on gesture production and yielded inconsistent findings. Some early studies claimed that patients with aphasia show the same deficits in gesture use as in speech production (Cicone et al., 1979; Glosser et al., 1986). More recent findings indicate that patients with aphasia are able to communicate better when they use gestures (Lanyon and Rose, 2009; Hogrefe et al., 2016; van Nispen et al., 2017). These more recent studies better controlled for other mediating factors like for co-occurring apraxia. Apraxia is a higher order motor disorder (Ochipa and Gonzalez Rothi, 2000; Goldenberg, 2008) that affects the ability to imitate gestures and to produce gestures on verbal command (Vanbellingen et al., 2010).

The perception of co-speech gestures seems to rely on neural networks implicated in language processing. Brain areas that are typically activated during language perception also respond when people perceive gestures (Andric and Small, 2012). This implies that co-speech gesture perception and language processing rely on shared neural networks (Xu et al., 2009). Indeed, it has been shown that multimodal integration of speech and gesture activates the left inferior frontal gyrus and the left middle temporal gyrus (for a comprehensive review see Dick et al., 2014; Özyürek, 2014). Moreover, the inferior frontal gyrus has shown to be more strongly activated when people perceive co-speech gestures than when they process speech without gestures (Kircher et al., 2009), or when they have to process mismatching gestures (Willems et al., 2007), or if they face gestures with higher levels of abstractness (Straube et al., 2011). Patients with aphasia typically show brain lesions to the perisylvian language network, thus their gesture processing might also be affected. Nevertheless, only few studies addressed gesture perception in aphasic patients (Records, 1994; Preisig et al., 2015; Eggenberger et al., 2016).

In healthy individuals, co-speech gestures presented on video or indirect interaction are fixated 0.5%–2% of the total viewing time (Gullberg and Holmqvist, 1999; Beattie et al., 2010). We found similar values for patients with aphasia viewing co-speech gestures on video (Preisig et al., 2015). However, gestures with a richer information content (i.e., meaning-laden gestures) show an increased probability of being fixated in healthy participants (Beattie et al., 2010). To date, it is still unknown whether or to which extent patients with aphasia attend to co-speech gestures during face-to-face interaction.

To answer this question, patients with aphasia and healthy controls were asked to participate in short conversations with an examiner, while their eye movements were recorded by means of a head-mounted eye-tracking system. At the same time, the conversations were filmed from a third-person perspective, in order to allow offline analysis of speech and co-speech gesture production. The advantage of conversational discourse is that it produces more ecologically valid results than presenting gestures on video displays or assessing gesture production by means of story narratives. Moreover, it has been shown that, under some circumstances, gestures presented in a face-to-face condition are more effective in conveying position and size information than those presented on video displays (Holler et al., 2009).

According to the classification proposed by Sekine et al. (2013), the produced gestures were either classified as meaning-laden gestures, which convey or indicate concrete meanings (e.g., iconic gestures representing object shapes), or abstract gestures, which convey abstract meaning (e.g., referential gestures assigned to entities in a narrative) or do not convey any specific meaning (e.g., repetitive movements timed with speech production). We expected that meaning-laden gestures would attract more visual fixations than abstract gestures. During face-to-face communication, information processing demands are higher, because patients have to take speaker turns, which is different from watching videos. Thus, we expected that patients may fixate gestures even more frequently than healthy participants, probably seeking additional nonverbal information.

Regarding gesture production, we expected that patients with aphasia produce more meaning-laden gestures than healthy participants as it has been reported by previous studies (Hadar et al., 1998a; Sekine et al., 2013), and that patients compensate for their reduced speech fluency by an increased gesture rate (Feyereisen, 1983). However, to the best of our knowledge, it has not yet been investigated whether lesions to specific brain areas are associated with an increased production of meaning-laden gestures in aphasia. Only few studies explored the neural substrates of spontaneous gesture production in clinical populations (Göksun et al., 2013, 2015; Hogrefe et al., 2017). The two studies by Göksun and colleagues focused on spatial aspects of gesture production in patients with right and left hemispheric brain lesions. Göksun et al. (2015) reported that patients with brain lesions involving the left superior temporal gyrus produce more gestures that illustrated the direction or the manner of movements (i.e., path gestures). Hogrefe et al. (2017) investigated the relationship between the comprehensibility of video retellings and patients’ lesions localization. Their findings suggest that lesions to left anterior temporal and inferior frontal regions play an important role for gesture comprehensibility in patients with aphasia. In the present study, we focused on assessing the production of meaning-laden gesture (in particular its frequency) in aphasia, using an ecologically valid paradigm with spontaneous speech, and aimed at linking this aspect with brain lesions by means of voxel-based lesion-symptom mapping (VLSM; Bates et al., 2003).

## Materials and Methods

### Participants

Twenty aphasic patients with first-ever unilateral stroke (mean age = 56.5, *SD* *±* 10.6 years; three women; 17 right-handed, two left-handed, one ambidexter) and 16 healthy controls (mean age = 58.7, SD ± 11.2 years; three women; 15 right-handed, one ambidexter) were included in the study. There were no group differences with respect to age (*t*_(34)_ = −0.625, *p* = 0.536), gender distribution ($\chi_{\left(1\right)}^{2}$ = 0.090, *p* = 0.764), handedness ($\chi_{\left(2\right)}^{2}$ = 1.702, *p* = 0.427), and education (*t*_(34)_ = −1.400, *p* = *0*.171). All participants had normal or corrected-to-normal visual acuity, and an intact central visual field of at least 30°. At examination, patients were in a sub-acute to chronic post-stroke state (1–68 months post-stroke, mean = 13.8, *SD* ± 20.0). Aphasia diagnosis was based on a standardized language assessment, performed by clinical speech-language therapists. Aphasia severity was assessed by means of the Aachen Aphasia Test (Huber et al., 1984). Concomitant apraxia was assessed using the standardized test of upper limb apraxia, TULIA (Vanbellingen et al., 2010). For an overview of patients’ individual clinical characteristics, see Table 1. Patients were recruited from three different neurorehabilitation clinics (University Hospital Bern, Kantonsspital Luzern, and Spitalzentrum Biel). This study was carried out in accordance with the recommendations of the local Ethics Committee of the State of Bern and of the State of Lucerne, Switzerland. The protocol was approved by the local Ethics Committee of the State of Bern and of the State of Lucerne. All subjects gave written informed consent in accordance with the Declaration of Helsinki.

**Table 1:** Individual clinical characteristics of the patient group.

Patient no.	Months post-onset	Etiology	Aphasia syndrome	Token Test (<91)	Comprehension (<92)	Written language (<91)	Naming (<93)	Repetition (<89)	TULIA (<194)	Lesion location
1	24	isch	Anomic	67	100	86	93	67	225	T, P
2	14	isch	Anomic	77	88	99	81	96	227	F, aARC
3	5	isch	Broca	63	41	57	47	49	179	F, T, aARC, BG, SC
4	1	isch	Anomic	95	100	93	89	83	224	F, T, SC
5	49	isch	Broca	74	65	86	n.a.	n.a.	197	F, aARC, SC
6	68	hem	global	30	45	31	n.a.	n.a.	194	F, aARC, BG, SC
7	3	isch	global	22	56	19	37	15	145	T, P, O, aARC, BG, SC
8	37	isch	Anomic	79	88	82	33	n.a.	189	T, P
9	1	isch	residual	97	100	93	100	99	216	F, aARC
10	2	isch	global	46	62	48	n.a.	n.a.	165	F, aARC, SC
11	44	isch	Wernicke’s	42	86	86	55	n.a.	153	T, P
12	3	isch	Broca	75	70	93	81	58	192	F, aARC, SC
13	8	isch	Broca	83	98	75	n.a.	n.a.	153	T, P
14	1	isch	Broca	72	65	82	59	94	181	right F, right SC, right BG
15	2	isch	Anomic	99	85	77	99	99	219	BG, SC
16	10	isch	Broca	93	98	58	74	47	186	F, T, aARC
17	1	isch	Anomic	89	92	75	86	77	209	T, P
18	2	isch	Wernicke’s	77	92	95	89	92	137	F, T, aARC
19	1	isch	Anomic	65	83	70	68	99	193	BG, SC
20	1	isch	Wernicke’s	76	53	89	42	53	176	T, P

*Note: Etiology: isch, ischemic infarction of medial cerebral artery, hem, hemorrhagic infarction (parenchyma bleeding); Percentile rank scores relative to the aphasia norm group in the different subtest of the AAT test battery (Token Test, Comprehension, Written Language, Naming and Repetition); TULIA (raw score): test of upper limb apraxia, in brackets cut off for pathological values (AAT and TULIA): Lesion location, F, frontal, T, temporal, P, parietal: O, occipital, BG, basal ganglia: SC, subcortical; aARC, anterior arcuate fasciculus*.

### Experimental Procedure

Participants were invited to take a seat on a chair without armrests. The examiner was sitting across the participant, in a slightly turned position. The distance between the participant and the experimenter was approximately 70 cm. The experiment began with an icebreaker conversation, during which the participants could familiarize themselves with the experimental situation. The main experiment, following thereafter, consisted of short conversations about four different topics of everyday life (favorite dish, habitation, leisure activities and education). The participants were told that they were going to participate in four conversations about given topics, which would each last 4–5 min. It was pointed out that they would not participate in an interview, but in a conversation, and they were encouraged to ask questions to the examiner at any time. In turn, the examiner also contributed to the interaction himself. The conversations were filmed with two cameras: one placed in the first-person perspective of the participant, i.e., the head-mounted eye-tracking scene camera, and the other placed in a third-person perspective, i.e., an additional camera (Sony HDR-CX570) that was fixed on a tripod. The additional camera captured a profile view including both interlocutors, i.e., the participant and the examiner.

### Eye-Tracking Data

During the main experiment, the eye movements of the participants were recorded using a head-mounted eye-tracking system (SMI HED, SensoMotoric Instruments GmbH, Teltow, Germany). This eye-tracking system has a temporal resolution of 50 Hz, a spatial resolution of typically <0.1°, and a tracking accuracy of typically 0.5–1°. The device consists of two cameras, which are fixed on a helmet. One camera records the scene from the perspective of the participant. In order to be able to capture the whole gesture space surrounding the examiner, this camera was equipped with a special lens (Lensagon BF2M15520), with a focal length of 1.5 mm and a field of view of 185°. The other camera captures the participant’s pupil and corneal reflection. The system was calibrated by means of a five-points calibration procedure. To ensure the accuracy of gaze position tracking over time, the calibration procedure was repeated prior to each conversation. Gaze direction was recorded from the right eye.

**Figure 1:**
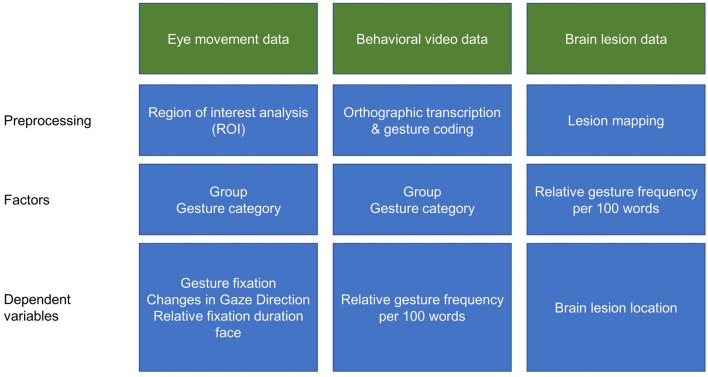
Schematic illustration of the data analyses procedures.

Pre-processing of eye fixation data was conducted with the BeGaze™ analysis software (SensoMotoric Instruments GmbH, Teltow, Germany). First, separate regions of interest (ROIs) were defined on a reference image of the examiner for the areas including hands, face, body and environment. Subsequently, individual fixations were mapped onto the corresponding position of the reference image, by means of the SMI Semantic Gaze Mapping analysis software tool (SensoMotoric Instruments GmbH, Teltow, Germany). The resulting output is represented by a data file, in which each fixation of each participant is associated with the corresponding ROI for a schematic illustration of the data analyses procedure see Figure 1.

### Analysis of the Behavioral Video Data

The analysis of behavioral video data was conducted with the freely available linguistic annotation software ELAN (Lausberg and Sloetjes, 2009). In ELAN, the videos from the two cameras (first-person perspective of the participant and third-person perspective on the conversational scene) were synchronized. For each conversation, an annotation time window of 90 s, positioned in the middle of the conversation, was selected for analysis. This led to a total of 6 min of behavioral video data analyzed per participant.

In a first step, the occurrence of speech and co-speech gestures during the conversation was segmented separately for the participant and the examiner. The occurrence of co-speech gestures was defined with respect to the stroke phase of the speech-accompanying gesture unit (Kendon, 2004), when the movement excursion is closest to its peak.

We did not expect a systematic difference in the behavior of the examiner between dyads including patients with aphasia and healthy participants. The dyads did not differ with respect to the number of turns taken by the examiner (*t*_(31.75)_ = −0.004, *p* = 0.997), the mean duration of the turns taken by the examiner (*t*_(31.61)_ = 0.512, *p* = 0.613), and the mean number of words per turn produced by the examiner (*t*_(33.50)_ = 0.154, *p* = 0.876).

Gesture classification was based on a coding scheme customized for the categorization of co-speech gestures in patients with aphasia (Sekine et al., 2013; see also Supplementary Material). This coding scheme mainly relies on the seminal gesture categories originally proposed by McNeil (1992), including the following gesture categories: *referential gestures, concrete deictic gestures, pointing to self, iconic observer viewpoint gestures* (OVPT), *iconic character viewpoint* (CVPT), *pantomime gestures, metaphoric gestures, emblems, time gestures, beats, letter gestures and number gestures* (for a comprehensive description of each individual gesture category see Sekine et al., 2013). The advantage of this classification system is that it also includes gesture categories more commonly observed in patients with aphasia (e.g., pointing to oneself). Moreover, most of these gesture categories have been used by previous studies (Cicone et al., 1979; McNeill, 1992; Gullberg and Holmqvist, 2006). Following the categorization described by Sekine et al. (2013), we assigned concrete deictic gestures, emblems, iconic CVPT gestures, iconic OVPT gestures, letter gestures, number gestures and pointing to self to the group of meaning-laden gestures, whereas beat gestures, metaphoric gestures, referential gestures, and time gestures were assigned to the group of abstract gestures. An overview on the relative gesture frequency per gesture category per 100 words is shown in Table 2. The gesture frequency of the examiner was analyzed by means of a repeated-measures analysis of variance (ANOVA) with the between-subjects factor group (aphasia; control) and the within-subjects factor gesture category. The analysis revealed neither a main effect of Group (*F*_(1,384)_ = 0.004, *p* < 0.952), nor an interaction Group × Gesture Category (*F*_(11,384)_ = 0.718, *p* < 0.721), indicating that there was no systematic group bias in the gesture behavior of the examiner.

**Table 2:** Relative gesture frequency per gesture category per 100 words (Standard deviations in parentheses).

	Participants		Examiner	
	Aphasia	Controls	Dyads with patients	Dyads with controls
*1) Meaning-laden gestures*				
Iconic CVPT	0.29 (0.49)	0.13 (0.21)	0.23 (0.22)	0.13 (0.15)
Iconic OVPT	0.50 (0.81)	0.17 (0.21)	0.77 (0.53)	0.60 (0.40)
Deictic	0.08 (0.18)	0.05 (0.10)	0.24 (0.27)	0.11 (0.13)
Emblem	0.42 (0.57)	0.18 (0.28)	0.16 (0.22)	0.13 (0.13)
Pantomime	0.08 (0.15)	0.03 (0.07)	0.02 (0.06)	0.01 (0.03)
Letter	0.13 (0.59)	0	0	0
Number	0.44 (0.96)	0.04 (0.12)	0.03 (0.08)	0.03 (0.13)
Pointing to self	0.20 (0.41)	0.01 (0.05)	0.13 (0.19)	0.06 (0.12)
*2) Abstract gestures*				
Referential	3.19 (3.36)	1.70 (1.63)	2.34 (0.62)	2.45 (0.69)
Beat	4.27 (3.91)	7.26 (4.57)	1.11 (0.67)	1.74 (0.76)
Metaphoric	0.02 (0.08)	0.01 (0.06)	0.07 (0.15)	0.06 (0.08)
Time	0.15 (0.43)	0.06 (0.15)	0.03 (0.06)	0.02 (0.06)

*Note: CVPT, character view point; OVPT, observer view point*.

To ensure the reliability of the gesture coding, 25% of the analyzed video data (i.e., one randomly selected conversation per participant) was coded by a second, independent rater. The percentage of agreement between the two raters was 86% on average for both groups (aphasia; control). Cohen’s kappa statistics were applied to determine the interrater reliability for the coding of gesture categories. The agreement between the two independent coders was high for both groups, patients with aphasia (kappa = 0.84) and healthy participants (kappa = 0.81), respectively. Any coding disagreement was resolved through discussion.

### Data Analysis

In a first step, pre-processed eye fixation data were extracted from the BeGaze™ analysis software, and behavioral data were extracted from the ELAN software, for processing with Matlab 8.0.0.783 (Mathworks Inc., Natick, MA, USA). Based on the event-related eye-tracking data, three dependent variables were calculated: the binomial variables: (1) overt gesture fixation (i.e., whether co-speech gestures produced by the examiner were fixated by the participant); (2) change in gaze direction during a respective gesture unit, as well as; (3) the relative fixation duration on the face area of the examiner. All variables were computed separately for meaning-laden and abstract gestures. Previous research showed that healthy participants are gazing only few times towards the gesturing hand (Gullberg and Holmqvist, 1999; Beattie et al., 2010). Therefore, changes in gaze direction during a respective gesture unit were considered as an additional measure of covert attention towards co-speech gestures. When the examiner produced a co-speech gesture and the participant fixated more than one ROI (hands, face, body, or environment), this was considered as a change in gaze direction. Furthermore, the individual speech fluency (i.e., the number of words per minute), and the frequency of gestures per 100 words, were calculated based on the behavioral video data.

Statistical analyses were conducted with the open-source program R (Ihaka and Gentleman, 1996). Two separate generalized linear mixed models (GLMM) with logit distribution were fitted for the binomial variables gesture fixation and change in gaze direction. Another GLMM with a Poisson distribution was fitted for the variables relative gesture frequency per 100 words per participant, including the covariate gesture frequency of the examiner as fixed effect in the model. Since the Poisson distribution includes only integer values, the absolute gesture frequency was taken as dependent variable in the GLMM and the number of words produced by every participant was modeled as an offset variable (Agresti, 2002), in order to account for the relative frequency of gestures per 100 words. A two-way repeated-measures ANOVA was calculated for the dependent variable relative fixation duration on the examiner’s face.

For *post hoc* comparisons, *p* values of individual contrasts were adjusted with the *Holm-Bonferroni* correction. For the patient subgroup, non-parametric Spearman correlations (two-tailed) were calculated between the dependent variable (relative frequency of gestures per 100 words) and the scores reflecting aphasia severity (mean percentile rank AAT), apraxia severity (TULIA) and speech fluency (words per minute), respectively.

### Lesion Mapping

Lesion analysis on imaging data was conducted with the open source software MRIcron (Rorden et al., 2012). MRI scans were available for 15 patients, and CT scans for the remaining five patients. Two patients were excluded from the lesion analysis for the following reasons: one patient was left-handed and had crossed-aphasia (i.e., aphasia due to a right-hemispheric stroke); another patient was ambidexter. If the MRI scans were obtained within 48 h post-stroke, diffusion-weighted MRI sequences were selected, otherwise FLAIR- or T2-weighted scans were used. For both MRI and CT scans, the lesions were delineated directly onto the transversal slices of the scans, resulting in a volume of interest (VOI) lesion file. The lesion VOI was then normalized into the Talairach space using the spatial normalization algorithm implemented in the MRICron clinical toolbox (Rorden et al., 2012), which is available for SPM8^1^. This toolbox provides templates that allow spatial normalization algorithms to be applied for both MRI and CT scans. VLSM was conducted in order to relate gesture production in aphasic patients to brain damage location. VLSM is a statistical analysis tool that allows to ascertain a direct relationship between brain tissue damage and behavior, on a voxel-by-voxel basis, in a comparable way as functional neuroimaging (Bates et al., 2003). We applied *t*-tests with family-wise error correction (FWE-corrected level at *p* < 0.05). Only voxels surviving a conservative permutation thresholding (4000 permutations) were considered. Voxels that were damaged in less than 20% of the patients were excluded from the analysis.

## Results

### Gesture Perception

**Figure 2:**
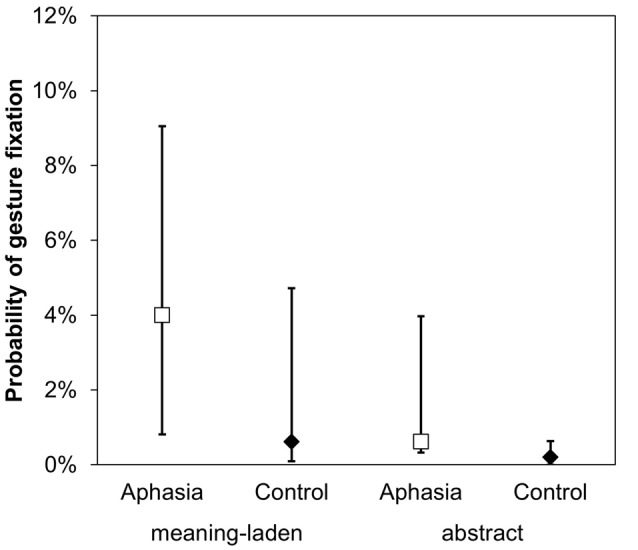
Gesture perception. An illustration of the main effects of gesture category and group on the dependent variables probability of overt gesture fixation. Error bars represent the 95% confidence intervals around the estimated values.

A GLMM including the fixed factors gesture category (meaning-laden; abstract) and group (aphasia; control) was calculated on the dependent variable overt gesture fixation (1 = fixation on the gesture; 0 = no fixation). As hypothesized, meaning-laden gestures were more frequently fixated than abstract gestures (*z* = 2.822, *p* = 0.002). Moreover, the analysis revealed a group effect, indicating that patients with aphasia were more likely to fixate the gestures produced by the examiner than healthy participants (*z* = −2.194, *p* = 0.014; Figure 2).

**Figure 3:**
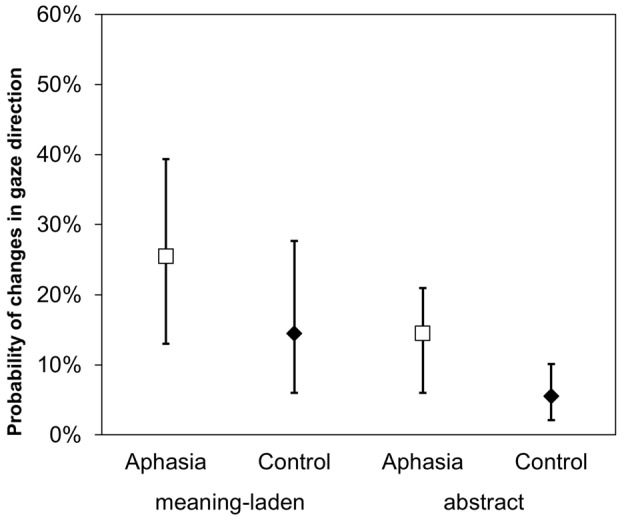
Changes in gaze direction. An illustration of the main effects of gesture category and group on the dependent variables probability of change in gaze direction. Error bars represent the 95% confidence intervals around the estimated values.

In line with the results obtained for the variable gesture fixation, a GLMM on the variable change in gaze direction showed a significant impact of the factors gesture category (*z* = − 4.684, *p* < 0.001) and group (*z* = −1.728, *p* = 0.044). Meaning-laden gestures led to more changes in gaze direction than abstract gestures, and patients with aphasia were more likely to change their direction of gaze during co-speech gestures than healthy participants (Figure 3).

A two-way repeated-measures ANOVA was computed for the dependent variable relative fixation duration on the face of the examiner, with the within-subjects factor gesture category (meaning-laden; abstract) and the between-subjects factor group (aphasia; control). The analysis revealed a significant main effect of the factor gesture category (*F*_(1,68)_ = 5.418, *p* < 0.026), but no significant effects of the factor group or of the interaction Group × Gesture Category. *Post hoc* comparisons revealed a statistical trend (*p* = 0.059) towards a lower fixation time on the face area for meaning-laden gestures (*M*_meaning-laden_ = 83.36%; *SD*_meaning-laden_ = 27.58%) compared to abstract gestures (*M*_abstract_ = 93.00; *SD*_abstract_ = 9.83%). This result may indicate that meaning-laden gestures draw more attention than abstract gestures, both in patients with aphasia and in healthy participants.

### Gesture Production

**Figure 4:**
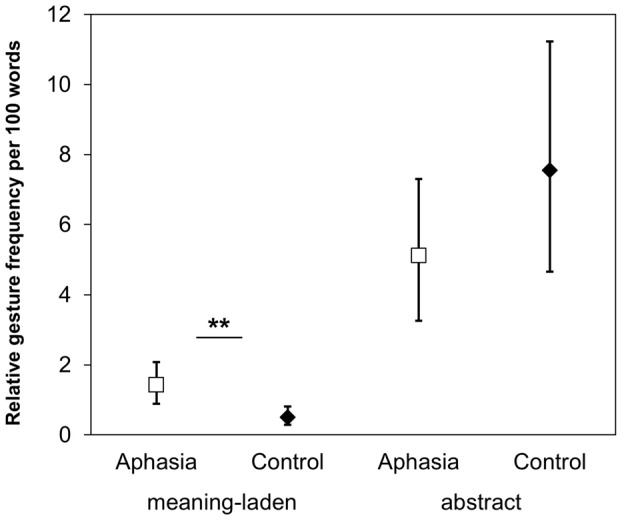
Gesture production. The relative gesture frequency per 100 words illustrating the Gesture Category × Group interaction. Asterisks denote the significant *post hoc* comparison (**p < 0.01). Error bars represent the 95% confidence intervals around the estimated values.

For the relative gesture frequency of co-speech gestures per 100 words, a GLMM including the fixed factors gesture category (meaning-laden; abstract) and group (aphasia; control) revealed a significant main effect of gesture category (*z* = −12.927, *p* < 0.001), and an interaction Gesture Category × Group (*z* = −7.757, *p* < 0.001). In both groups, the participants produced more abstract gestures than meaning-laden gestures (*p* < 0.001). *Post hoc* comparisons revealed that the relative frequency of meaning-laden gestures was higher in patients with aphasia than in healthy participants (*p* = 0.006), but there was no group difference regarding abstract gestures (*p* = 0.374; see Figure 4).

In order to determine the influence of the factors aphasia severity, speech fluency, month post-stroke and apraxia severity in the patient subgroup, correlation analyses were conducted between these variables and the relative gesture frequency per 100 words. We found that patients with more severe aphasia (as reflected by lower scores on the AAT) produced more meaning-laden gestures (*r*_s_ = −0.58, *p* = 0.008). Speech fluency was negatively correlated with the relative frequency of abstract (*r*_s_ = −0.64, *p* = 0.002) and meaning-laden gestures (*r*_s_ = −0.57, *p* = 0.009), respectively. There was no significant correlation between the duration in months post-stroke and gesture production. In addition, we compared the two patient subgroups (sub-acute vs. chronic): 1–2 months post-stroke (sub-acute), 3–68 months (chronic). We did not find a significant difference between the sub-acute (*Md* = 1.26, 95% *CI* (0.50 2.39)) and chronic patients (*Md* = 0.88, 95% *CI* (0.65 4.81)) for the production of meaning-laden gestures (Mann Whitney U-Test, *Z* = 0.684, *p* = 0.503). Apraxia severity (as assessed as an overall index and on different subscales for imitation and pantomime of non-symbolic (meaningless), intransitive (communicative) and transitive (tool-related) hand movements) was not correlated with frequency of spontaneous co-speech gestures. Therefore, we will not further elaborate on the potential influence of apraxia in the course of this article. Previous studies reported an influence of aphasia syndrome on gesture production (Sekine and Rose, 2013; Sekine et al., 2013). Our patient group included seven Anomic (*Md* = 0.660, 95% *CI* (0.33, 1.57)), six Broca (*Md* = 2.06, 95% *CI* (0.10, 6.31)), three Wernicke’s (*Md* = 1.28. 95%, *CI* (−1.70, 5.52)), three global (Md = 3.01, CI (−6.75, 14.34)), and one patient with residual aphasia. We did not find a significant group differences with regards to the relative frequency of meaning-laden gestures (Kruskal-Wallis Test, *Z* = 3.625, *p* = 0.305).

### Lesion Analysis

**Figure 5:**
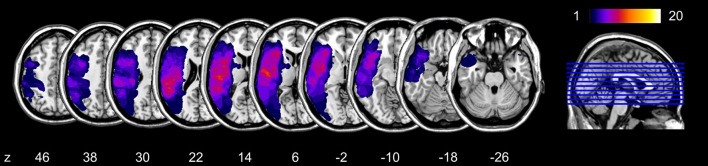
Overlap maps of the brain lesions in the patient group. The z-position of each axial slice in the Talairach stereotaxic space is presented at the bottom of the figure.

**Figure 6:**
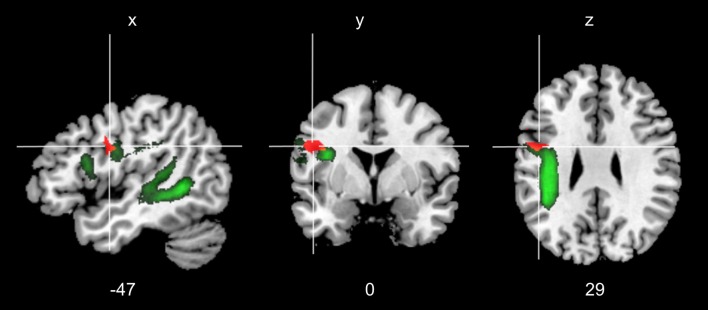
Voxel-based lesion-symptom mapping (VLSM). Voxels depicted in orange represent brain damage locations that were significant predictors of an increased frequency of meaning-laden gestures per 100 words (FWE-corrected level at *p* < 0.05). The left arcuate fasciculus is represented in green, based on a recently published probabilistic DTI atlas (threshold >50%, de Schotten et al., 2011). Talairach coordinates of the center of mass are presented at the bottom of the figure.

The overlay of the patients’ individual cerebral lesions is shown in Figure 5. The mean volume of individual brain lesions in patients with aphasia was 43.20 cm^3^ (*SD* = 35.99 cm^3^). The lesion VLSM analysis aimed to identify brain tissue damage that is associated with an increased production of meaning-laden gestures during face-to-face interaction. The VLSM model included the variable relative frequency of meaning-laden gestures and it revealed a significant lesion cluster (FWE-corrected level at *p* < 0.05) on the anterior end of the arcuate fasciculus (Talairach coordinates center of mass; −47, 0, 29; Figure 6).

## Discussion

The main question of this study concerned how patients with aphasia perceive and produce co-speech gestures during face-to-face interaction. We studied the impact of co-speech gestures at the level of perception by means of eye movement recordings, and we applied VLSM in order to relate co-speech gesture production with brain injury localization. We found that meaning-laden gestures are more likely to attract visual attention than abstract gestures, and that patients with aphasia are more likely to fixate co-speech gestures than healthy participants. Regarding gesture production, patients with more severe aphasia, but not with more severe apraxia, produced more meaning-laden gestures than patients who were mildly affected. Finally, brain lesions involving the anterior part of the arcuate fasciculus are related to an increased production of meaning-laden gestures in patients with aphasia.

In accordance with the results of previous studies (Gullberg and Holmqvist, 1999; Beattie et al., 2010; Preisig et al., 2015; Eggenberger et al., 2016), patients with aphasia and healthy participants fixated the examiner’s face more often than his co-speech gestures. More relevantly, and according to our hypothesis, we found that meaning-laden gestures were significantly more often fixated and elicited more changes in gaze direction than abstract gestures. This finding corresponds well with results reported in healthy participants (Beattie et al., 2010), showing that gestures with a higher information content are more frequently fixated. Co-speech gestures seem also to modulate gaze direction towards the gesturing hands of the speaker, as indicated by more frequent changes in gaze direction, and a reduced fixation time on the speaker’s face area. Our results show, for the first time, that meaning-laden gestures attract more visual attention than abstract gestures, and that patients with aphasia fixated co-speech gestures more frequently than healthy participants. This finding, obtained in a face-to-face interaction setting, implies that patients may benefit from multimodal information provided by meaning-laden gestures, as suggested by previous findings obtained during video observation (Eggenberger et al., 2016). Beyond, the finding strengthens the importance of nonverbal communication in comprehension of speech acts (Egorova et al., 2016).

In contrast to the results of our previous reports (Preisig et al., 2015; Eggenberger et al., 2016), we found that patients with aphasia fixated co-speech gestures of their interlocutor with a higher probability than healthy participants. Furthermore, patients with aphasia changed their direction of gaze more frequently than healthy participants in reaction to gestures made by the examiner. In comparison to video observation, face-to-face interaction imposes additional demands, such as taking turns while speaking, which require the planning and initiation of own speech acts (Pickering and Garrod, 2013). In aphasia, lexico-syntactic processing with regard to language comprehension and speech production is impaired (Caplan et al., 2007). This means that, with increasing complexity of the speech content, the detection of a relevant time point for turn transition becomes more difficult for patients with aphasia (Preisig et al., 2016). Therefore, it is conceivable that co-speech gestures gain higher relevance in aphasia, because face-to-face interactions impose higher task demands on aphasic patients than on healthy participants.

Regarding gesture production, we found that aphasic patients produced significantly more meaning-laden gestures, but not more abstract gestures, compared to healthy participants. Together with recent studies (Lanyon and Rose, 2009; Hogrefe et al., 2016; van Nispen et al., 2017), our findings indicate that patients with aphasia are able to produce gestures in order to communicate information. In a recent study, Hogrefe et al. (2016) compared the production of meaning-laden hand movements during story narratives in patients with left or right brain damage, and in healthy participants. The authors found that, in comparison to healthy participants, patients with left-hemispheric lesion showed an increased use of meaning-laden hand movements, whereas patients with right-hemispheric lesion showed a decreased use of these gestures. The reported findings contradict the notion of a parallel impairment of verbal and gestural abilities in aphasia (McNeill, 1992; Kendon, 2004).

Our results also reveal a significant impact of aphasia severity and speech fluency on gesture production. Patients with more severe aphasia produced significantly more meaning-laden gestures. Patients with reduced speech fluency produced both more meaning-laden and more abstract gestures. Similar associations were reported during free conversation (Feyereisen, 1983), and during personal narrative interviews in patients with aphasia (Sekine et al., 2013). There are two possible interpretations of the reported findings. First, the more severely affected patients may produce more meaning-laden gestures as a nonverbal compensation strategy for their language deficits (Behrmann and Penn, 1984; Hogrefe et al., 2013). Second, beyond their communicative meaning, co-speech gestures may also facilitate lexical retrieval, as reported in healthy participants (Krauss and Hadar, 1999) and in patients with aphasia (Hadar et al., 1998a,b). Therefore, the relationship between speech fluency and gesture frequency may alternatively be explained by the facilitating effect of gesturing on speech production (Hadar et al., 1998a,b).

Finally, VLSM revealed that patients who produced more meaning-laden gestures significantly more often showed a brain lesion involving the anterior part of the arcuate fasciculus, in close vicinity of the precentral gyrus. The lesion cluster spares areas which has been associated with gesture and speech integration in the lateral temporal lobe and the inferior frontal gyrus (Andric and Small, 2012). The arcuate fasciculus is a white matter tract that connects Wernicke’s area with Broca’s area. Patients with lesions to the arcuate fasciculus usually display heterogeneous symptoms, depending on the exact location of the lesion (Levine and Calvanio, 1982). Findings obtained from diffusion-tensor imaging demonstrated that the complexity of the structure of the white matter tract may account for the heterogeneity of the symptoms resulting from its lesion (Catani and ffytche, 2005). The arcuate fasciculus consists of three segments: first, direct pathway connects Wernicke’s area, in the left superior temporal lobe, and Broca’s area, in the left inferior frontal lobe; second, anterior segment connects Broca’s area with the parietal cortex; and third, posterior segment links the parietal cortex with Wernicke’s area. Recently, it has been shown that patients with aphasia show dissociable syndromes depending on affected arcuate segment (Yourganov et al., 2015). Brain lesions involving the anterior and the long segment of the arcuate fasciculus were found to be associated with slow and agrammatic speech production (i.e., Broca’s aphasia), whereas lesions to the posterior segment were found to be associated with sensory-motor language deficits (i.e., conduction aphasia). These findings, suggest that the integrity of the anterior part of the arcuate fasciculus is important for speech production, i.e., articulation. Interestingly, patients with brain lesions to the anterior arcuate fasciculus did not produce fewer co-speech gestures, as the assumption of a parallel impairment of speech and gesture production would imply (Cicone et al., 1979; Glosser et al., 1986). To the contrary, patients with brain lesion to the anterior arcuate produced more meaning-laden gestures than patients without a lesion to this area. From this observation, we conclude that these patients may produce more meaning-laden gestures, in order to compensate their speech production deficits. Moreover, the described lesion cluster also includes parts of the premotor cortex. The theory of embodied cognition proposes that semantic meaning is represented in the cortex depending on its modality (e.g., motor cortex would be involved in the processing of action-related semantics, like the processing of action-words; Pulvermüller, 2013). In the context of the current patient sample, we speculate that lesions to the premotor cortex and the anterior arcuate fasciculus could affect the mapping of meaning-to articulation and/or lexical semantic processing of action-related words, whereas the mapping of meaning to spontaneous gesture is relatively spared. In line with this hypothesis, it has been shown that the integrity of the upper limbs as measured by motor-evoked potentials is an important predictor for aphasia recovery (Glize et al., 2018). This might also indicate that the use of spontaneous co-speech gestures could have a predictive value for aphasia recovery.

One limitation of the current study is that cross-sectional data from patients in a sub-acute to chronic state do not allow to test the impact of gesture production on aphasia recovery conclusively. It has to be assumed that there are big differences in the individual inclination to produce co-speech gestures. Another limitation is that the applied VLSM approach tested the integrity of the arcuate fasciculus indirectly. In contrast, other approaches like diffusion tensor imaging would allow to estimate the integrity of the left arcuate fasciculus on an individual participant level. Finally, an assessment of post-stroke depression could have been informative in order to exclude a confounding influence of the patients’ affective state on their gesture production.

## Conclusion

The present study showed that patients with aphasia, as healthy participants, attend more often to meaning-laden gestures than to abstract gestures in a face-to-face dialog situation. Overall, aphasic patients fixated co-speech gestures more frequently than healthy participants. This suggests that patients with aphasia may benefit from the perception of multimodal information provided by co-speech gestures. This notion is supported by the fact, that patients with aphasia fixated meaning-laden gestures more frequently than abstract gestures. In contrast to gesture perception, there was a relation between aphasia severity and gesture production. Patients with more severe aphasia and reduced speech fluency produced more meaning-laden gestures. Moreover, an increased production of meaning-laden gestures was associated with brain lesions to the anterior arcuate fasciculus. This area is supposed to be related to speech production abilities. Therefore, we conclude that patients with aphasia produced more meaning-laden gestures, to compensate for their verbal production deficits. These findings have implications for aphasia therapy and for the patients’ daily interactions with their family members, because they suggest that patients with aphasia can use meaning-laden gestures as alternative means of communication. This means that rehabilitation professionals should increase the awareness of potential interlocutors for the gestures produced by people with aphasia.

## Author Contributions

RM, BP, NE, TN and J-MA: designed research. BP and NE: performed research. DC, KG, TN and JM: contributed new reagents or analytic tools. BP: analyzed the data. BP, RM, DC and NE: wrote the article.

## Conflict of Interest Statement

The authors declare that the research was conducted in the absence of any commercial or financial relationships that could be construed as a potential conflict of interest.

## Footnotes

^1^http://www.fil.ion.ucl.ac.uk/spm/
