# Supplementary material for: Multimodal Communication in Aphasia: Perception and Production of Co-speech Gestures During Face-to-Face Conversation
Source: Front Hum Neurosci. 2018 Jun 14;12:200. doi: 10.3389/fnhum.2018.00200 (PMC6010555; doi:10.3389/fnhum.2018.00200)
Supplement: Supplementary file 1 [file Data_Sheet_1.docx]

## Appendix

Table 3: Gesture categories (Sekine & Rose, 2013; Sekine et al., 2013), table adapted from van Nispen et al. (2017).

| Type | | Description |
| --- | --- | --- |
| *1) Meaning-laden gestures* | |  |
|  | Iconic character viewpoint (CVPT) | Uses the speaker’s own body in depicting a concrete action, event, or Object, as though he is the character/Object itself. For example, to depict someone running, he swings his arms back and forth, as if he is running. |
|  | Iconic observer viewpoint (OVPT) | Depicts a concrete action, event, or Object as though the speaker is observing it from a far. For example, to depict someone running, the speaker traces her index finger in the frontal space from left to right as if she is seeing the scene as an observer. |
|  | Deictic | Indicates a concrete referent in the physical environment, such as a picture book or an item of actual clothing. |
|  | Emblem | Form and meaning are established by the conventions of specific communities and can usually be understood without speech, such as thumb and pointer finger making a circle Shape for OK. |
|  | Pantomime | Consists of two or more CVPT gestures, which occur continuously within the same gesture unit. No matter how many CVPT gestures occur continuously, they are counted as one pantomime. |
|  | Letter | Movements associated with writing letters in the air or on the desk or on one’s thigh with an empty hand or fingers. |
|  | Number | Uses the speaker’s fingers to display numbers. |
|  | Pointing to self | The speaker points to his or her own body (mostly the chest) in order to refer to him- or herself. |
|  |  |  |
| *2) Abstract gestures* | |  |
|  | Referential | Is used to assign the entity of referents, such as Objects, places, or characters in the story, into the space in front of a speaker where any concrete Object is absent. The hand Shape of the gesture usually takes the form of a pointing gesture or of holding some entity. Is used to assign the entity of referents, such as Objects, places, or characters in the story, into the space in front of a speaker where any concrete Object is absent. The hand Shape of the gesture usually takes the form of a pointing gesture or of holding some entity. |
|  | Beat | Movements that do not present a discernible meaning and are recognized by their prototypical repetitive movement characteristics timed with speech production. |
|  | Metaphoric | Presents an image of an abstract concept, such as knowledge or justice, language itself, the genre of the narrative, and so on. It often has a cup-Shaped hand Shape. |
|  | Time | Indicates some space to denote a time, such as past (back of the body) or future (front of the body). |
| Note: These gesture types were not further analyzed in the present study. | | |
